# MCL-YOLO: A Multi-Module Collaborative Lightweight Object Detection Method for Bridge Crack Detection

**DOI:** 10.3390/s26185801

**Published:** 2026-09-13

**Authors:** Bingyu Han, Yang Wu, Wenhao Feng, Xiaoman Mi

**Affiliations:** 1 Transportation Institute, Inner Mongolia University, Hohhot 010070, China; byhan@imu.edu.cn (B.H.); 15500199296@163.com (Y.W.); 15234761073@163.com (X.M.); 2 Inner Mongolia Key Laboratory of Structural Testing for Colleges and Universities, Inner Mongolia University, Hohhot 010070, China

**Keywords:** bridge crack detection, MCL-YOLO, lightweight object detection, receptive-field attention, dynamic feature enhancement

## Abstract

Bridge surface cracks are important early indicators of structural performance degradation. However, affected by complex environmental interferences and irregular morphologies, existing models still fall short in micro-crack recognition, accurate bounding-box localization, and lightweight. To address these challenges, this study proposes a multi-module collaborative lightweight model (MCL-YOLO) based on YOLOv12. Specifically, the existing ADown module from YOLOv9 is incorporated into the YOLOv12 architecture to reduce computational complexity while preserving critical information during feature downsampling. To enhance the representation of slender, curved, and branched crack patterns, a C3k2-RFAConv module is designed by integrating a receptive-field attention mechanism. Furthermore, an iEMA module is embedded before the high-resolution detection branch to strengthen the semantic response to weak-texture cracks. A bridge crack dataset containing 4029 images was constructed to evaluate the proposed model. Experimental results show that MCL-YOLO achieves Precision, Recall, *mAP*@50, and *mAP*@50:95 values of 0.891, 0.757, 0.844, and 0.675, with 5.5 GFLOPs, 2.240 M parameters, and a model-file size of 4.689 M. Compared with the YOLOv12n baseline, MCL-YOLO improves the four detection metrics by 2.30%, 4.56%, 4.07%, and 3.21%, while reducing GFLOPs and parameter count (Params) by 12.70% and 12.77%, respectively. Ablation experiments, model version comparisons, attention mechanism comparisons, and qualitative detection results collectively verify the effectiveness of the integrated architectural modifications.

## 1. Introduction

Bridges are a critical component of national transportation infrastructure and play a central role in facilitating the movement of people and goods, promoting regional integration, and driving economic growth [1]. By the end of 2023, the number of highway bridges in China had reached 1.0793 million, with a total length of 95.2882 million linear meters. Many of these structures have entered the middle and late stages of service, making performance degradation and aging increasingly prominent issues [2]. Under the coupled effects of material aging, environmental erosion, and load-induced fatigue, bridge surface cracks commonly appear as one of the most intuitive and common early manifestations of structural damage [3]. Cracks not only weaken the sectional stiffness and load-bearing capacity of structures but also provide physical pathways for the ingress of aggressive media, which may induce reinforcement corrosion and concrete deterioration and thereby seriously threaten structural durability and service life [4]. Therefore, early and accurate identification of bridge surface cracks has become a fundamental requirement for structural health monitoring and for ensuring the life-cycle safety of infrastructure [5]. Traditional bridge crack detection mainly relies on manual visual inspection and experience-based interpretation. However, this approach generally suffers from low efficiency, strong subjectivity, and poor repeatability, making it difficult to meet the demand for large-scale and rapid screening of modern bridge networks [6].

In recent years, with the rapid development of deep learning and computer vision, data-driven methods based on deep learning have provided a new pathway for bridge surface crack identification [5,7,8]. In particular, the YOLO series has emerged as a focal point of research due to its end-to-end inference architecture and favorable balance between detection accuracy and computational efficiency [9,10]. From the multi-scale feature pyramid of YOLOv3 [11], to the introduction of programmable gradient information in YOLOv9 [12], and further to the attention-centric YOLOv12 framework [13], which further improves the upper bound of real-time detection performance through enhanced attention mechanisms and residual connections, continuous optimization of model architectures has significantly improved general object detection performance. However, general object detection models still face severe challenges when applied to specific targets such as bridge cracks. Crack objects usually exhibit high aspect ratios, weak boundaries, and sparse texture information. When processing such targets, the original attention mechanism of YOLOv12 tends to suffer from dispersed attention responses, making it difficult to focus accurately on crack edges and weak-texture regions. In addition, conventional convolutions assign fixed contributions to spatial elements within each local receptive field, which limits their ability to emphasize feature responses associated with curved, branched, and irregularly extended crack morphologies. Furthermore, conventional downsampling operations are likely to cause the loss of critical structural information during feature compression, thereby weakening the model’s representation capability for tiny cracks and discontinuous edge cracks. Meanwhile, in edge computing scenarios, a significant trade-off persists between computational load and detection accuracy.

To address these limitations, this study proposes a Multi-Module Collaborative Lightweight YOLO (MCL-YOLO) algorithm, using YOLOv12 as the baseline architecture. The proposed algorithm integrates the iEMA dynamic feature enhancement module [14,15], which utilizes a multi-scale spatial interaction mechanism to strengthen the edge response of cracks with weak textures, thereby mitigating the loss of fine-grained details typical of general-purpose models. Meanwhile, the receptive-field attention-weighting mechanism of Receptive-Field Attention Convolution (RFAConv) is embedded into the C3k2 architecture to construct the C3k2-RFAConv composite feature extraction module [16]. Instead of predicting spatial offsets or changing sampling positions, this module adaptively reweights the contributions of spatial elements within each fixed local receptive field according to local feature responses, thereby enhancing the representation of elongated, curved, and branched crack patterns. To reduce computational complexity, the existing ADown module originally introduced in YOLOv9 is incorporated into the YOLOv12 architecture as a lightweight downsampling operator [12]. This integration optimizes the feature transmission path and improves the retention of critical information during downsampling. MCL-YOLO is designed to balance crack-feature enhancement, irregular-morphology adaptation, and a lightweight model footprint. This study provides a feasible technical solution for bridge crack detection and offers a reference for integrating computer vision with structural health monitoring. The main contributions of this study are summarized as follows:

(1) Considering the slender shapes, weak edges, sparse textures, and irregular morphologies of crack targets, this study proposes a multi-module collaborative lightweight detection framework (MCL-YOLO). This framework enhances detection accuracy while minimizing computational costs, thereby improving the model’s suitability for practical structural inspection.
(2) By introducing the iEMA dynamic feature enhancement module and the C3k2-RFAConv composite feature extraction module, this study constructs a feature extraction system designed for weak-edge and irregularly shaped cracks. Based on their respective cross-spatial interaction and receptive-field attention-weighting mechanisms, these modules are intended to improve feature representation for crack-edge details, slender structures, and irregularly extended morphologies.
(3) The ADown module is incorporated into the YOLOv12 architecture as a lightweight downsampling operator. This integration optimizes the feature transmission path while reducing FLOPs and alleviating the loss of critical information during downsampling.

## 2. Literature

Cracks serve as critical indicators of performance degradation in bridge and road infrastructure. Timely and accurate identification of crack locations and morphological characteristics is of great significance for structural health monitoring, damage early warning, and maintenance decision-making. Early crack detection mainly relied on manual inspection, image threshold segmentation, edge detection, morphological processing, and traditional machine learning methods. Abdel-Qader et al. [17] employed Fourier transform and Hough transform algorithms to analyze crack edge information and used the Canny operator to segment cracks and extract edge features. Vivekananthan et al. [18] combined grayscale discrimination with the Otsu method and successfully detected target cracks in different images. Although these methods have the advantages of simple implementation and strong interpretability, they are prone to broken crack extraction, false detections, and missed detections under uneven illumination, complex background textures, large crack-scale variations, and obvious noise interference [19,20]. Therefore, they are difficult to apply to large-scale, automated, and real-time detection in engineering field scenarios [21]. With the development of deep learning, crack detection algorithms based on object detection have become mainstream. Object detection methods can generally be divided into two-stage and one-stage algorithms. Two-stage detectors, such as Faster R-CNN and Mask R-CNN [22,23], rely on the generation of bounding boxes that may contain objects, followed by feature extraction and classification using CNNs. However, such models usually involve high computational complexity and impose relatively high requirements on annotation accuracy and training samples. As a result, their deployment cost is relatively high in scenarios such as edge devices and UAV-based inspection.

In contrast, one-stage models such as the YOLO (You Only Look Once) series have been widely adopted for crack detection because they can achieve accurate real-time detection. As illustrated in Table 1, the evolution of the YOLO series demonstrates that its main development trajectory has gradually shifted from an end-to-end one-stage detection framework toward efficient multi-scale feature fusion, lightweight, and attention-centric modeling. Subsequent versions, including YOLOv3, YOLOv7, YOLOv8, YOLOv9, and YOLOv10, have improved detection performance from different perspectives, such as multi-scale prediction, feature reuse, decoupled detection heads, programmable gradient information, and NMS-free training strategies. Redmon and Farhadi introduced the Darknet-53 backbone and multi-scale feature pyramid network (FPN) in YOLOv3 [11], which significantly enhanced the model’s adaptability to multi-scale targets and established an early benchmark for industrial defect detection. Li et al. subsequently developed YOLOv6 as a single-stage object detection framework for industrial applications [24]. Later, Wang et al. proposed the efficient layer aggregation network (E-ELAN) and re-parameterization techniques in YOLOv7 [25], greatly improving feature reuse efficiency through a decoupled design between training and inference. To address information attenuation and gradient vanishing in deep network training, Wang et al. introduced the programmable gradient information (PGI) mechanism and the GELAN architecture in YOLOv9 [12], providing a new perspective for performance optimization in deep detection networks. In YOLOv12 [13], long-range dependency modeling is further strengthened through an attention-centric architecture and an efficient area attention mechanism. The YOLO series has become one of the most widely used frontier frameworks for the intelligent screening of infrastructure damage.

Existing studies have mostly improved frameworks such as YOLOv4, YOLOv5, YOLOv7, YOLOv8, YOLOv10, and YOLOv11 by introducing feature fusion, attention mechanisms, multi-scale detection heads, and improved loss functions, thereby enhancing crack detection accuracy and real-time performance [26,27,28]. Su et al. [29] proposed MOD-YOLO, which reconfigures the feature information flow to achieve a balance between detection efficiency and accuracy. In terms of attention mechanisms and feature enhancement, Liu et al. [30] introduced R-YOLO v5, employing attention mechanisms and an optimized loss function to enhance detection precision. Similarly, Wang et al. [31] incorporated Vision Transformer into YOLOv5 to strengthen the global semantic representation of asphalt cracks, while Ye et al. [32] improved YOLOv7 by integrating three custom modules to better distinguish concrete cracks from confusing background targets. Furthermore, Liu et al. [33] proposed an improved YOLOv8 that fuses multi-scale attention mechanisms to enhance detection performance across varying crack scales. Zhang et al. [34] developed YOLOv10-DECA, utilizing decoupled enhancement and attention modeling to boost real-time detection accuracy for concrete cracks. Ding et al. [35] proposed SCD-YOLO, which incorporates cross-scale attention fusion and dynamic detection heads to refine localization for complex crack morphologies. Xing et al. [36] introduced EMG-YOLO, which improves performance on edge devices through decoupled detection heads, MPDIoU, and global context modeling. These methods have improved crack recognition capability in complex backgrounds to a certain extent. However, some attention modules introduce additional parameters and computational overhead, and their adaptability to the edge continuity and morphological variations of slender cracks remains limited.

Lightweight design and engineering deployment have become important development trends in crack detection research. Crack detection models need to maintain high recognition accuracy while achieving low computational complexity, a small parameter scale, and fast inference speed [37]. Existing studies mainly reduce model deployment costs through lightweight backbone networks, depthwise separable convolutions, channel compression, network pruning, and knowledge distillation. Lin et al. [38] proposed YOLO-ROC for road damage detection, enhancing multi-scale feature extraction via the BMS-SPPF module and implementing a hierarchical channel compression strategy to lower computational complexity. Xu et al. [39] developed an instance segmentation model for concrete cracks based on YOLOv8-seg. By integrating improved feature extraction modules, network pruning, and knowledge distillation, their model significantly reduced the number of parameters and computational cost while maintaining detection and segmentation accuracy. Liu et al. [40] proposed a YOLOv5-lite model for tunnel lining crack detection, which reduces computational requirements through EIoU loss optimization, network pruning, and knowledge distillation, while improving inference speed with high detection accuracy. Dong et al. [41] proposed the LPDD-YOLO model, which employs FasterNet for lightweight feature extraction and compensates for the accuracy loss caused by lightweight design through attention-based downsampling and deformable convolution. Zhang et al. [42] replaced the feature extraction backbone of YOLOv4 with a lightweight network, effectively reducing the number of model parameters and storage requirements while improving the real-time inference capability for bridge crack detection. Although existing studies have made progress in model compression and edge-device deployment, lightweight architectures may still weaken shallow detail representation, leading to false detections and missed detections of tiny cracks, weak-texture cracks, and cracks in complex backgrounds.

Existing crack detection research has evolved from traditional image processing methods to real-time object detection methods represented by YOLO, and has gradually progressed toward multi-scale feature fusion, attention enhancement, lightweight deployment, and robust detection in complex scenarios. However, current methodologies still face significant challenges in bridge crack detection: (1) severe degradation of edge information for slender and micro-cracks during downsampling; (2) limited adaptability of conventional convolutional operators to irregular crack morphologies; and (3) the difficulty in balancing computational efficiency with the preservation of edge details, multi-scale semantics, and complex structural representations in lightweight models. To address these issues, this study builds upon the YOLOv12 baseline by integrating ADown, C3k2-RFAConv, and iEMA modules. These modules collaboratively optimize the model in terms of sampling fidelity, receptive-field feature weighting, and critical semantic enhancement, thereby achieving a favorable balance between detection accuracy under the background conditions represented in the current dataset and theoretical computational complexity.

## 3. Methods

### 3.1. YOLOv12 Baseline Architecture

**Figure 1:**
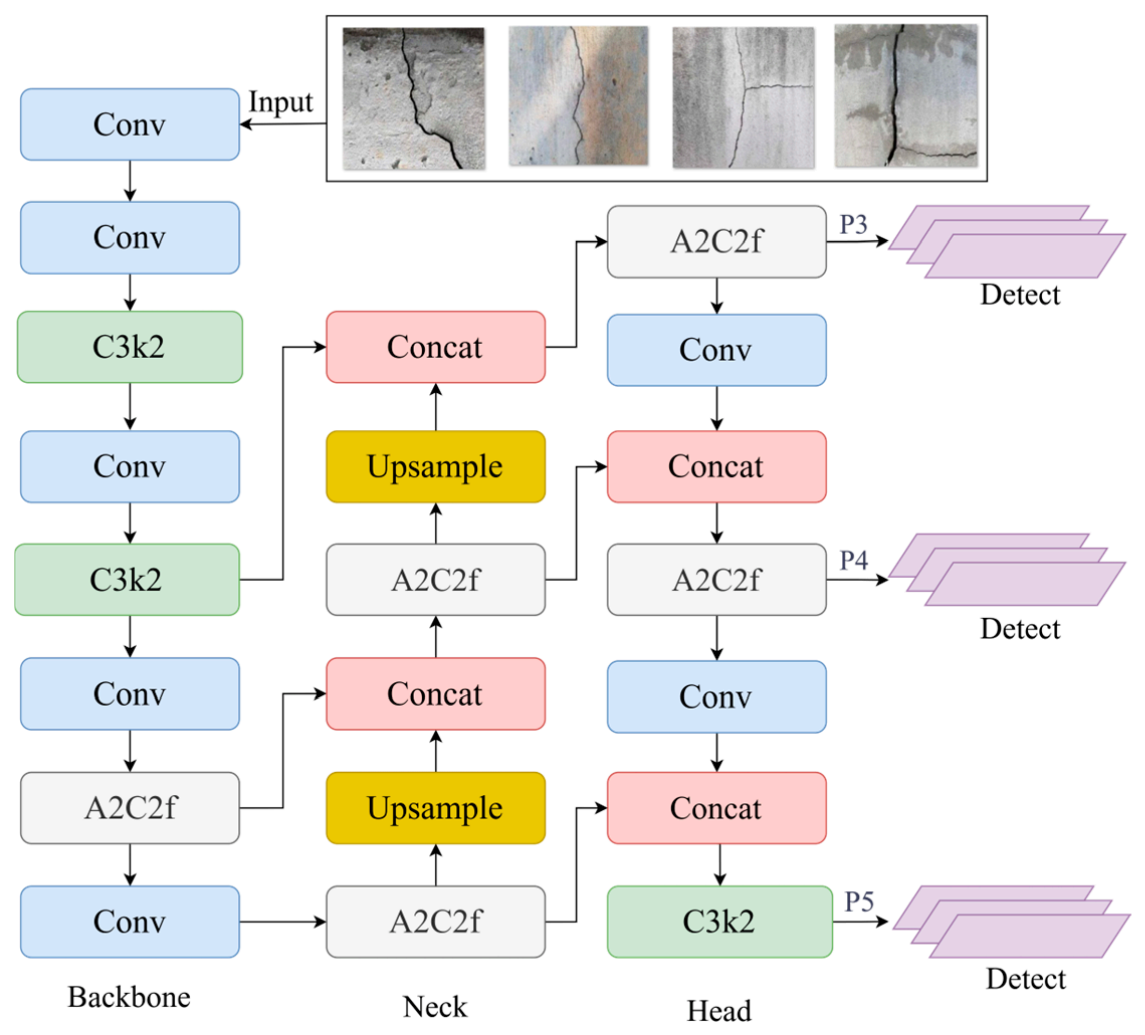
Overall architecture of YOLOv12.

YOLOv12 is a single-stage detection framework designed for real-time object detection. As illustrated in Figure 1, its overall architecture consists of three main components: the Backbone, Neck, and Prediction Head. The Backbone is responsible for progressively extracting low-level edge and texture features as well as high-level semantic information from the input image. The Neck performs cross-scale feature fusion through top-down and bottom-up information pathways, thereby enhancing feature interaction and semantic representation across different levels. The Prediction Head predicts object classification confidence and bounding box locations on the P3, P4, and P5 feature maps, thereby accommodating objects of different scales. Compared with conventional convolution-based detection networks, YOLOv12 introduces an attention-enhanced architecture while maintaining high inference speed and deployment efficiency, thereby improving long-range dependency modeling and the ability to focus on task-relevant regions [13]. Its key improvements are mainly reflected in the R-ELAN backbone feature extraction structure and the area attention mechanism. Specifically, R-ELAN improves feature aggregation and optimization stability by introducing block-level residual connections and a redesigned aggregation structure. Area Attention partitions the feature map into several regions along the horizontal or vertical direction, which reduces the computational cost of self-attention while preserving a relatively large effective receptive field. Furthermore, YOLOv12 incorporates modules such as A2C2f and C3k2, together with a multi-scale path aggregation strategy, during feature extraction and fusion. These designs further strengthen cross-scale feature interaction and spatial contextual modeling, allowing the framework to achieve a favorable balance among detection accuracy, inference speed, and model complexity in general object detection tasks.

### 3.2. MCL-YOLO Architecture

**Figure 2:**
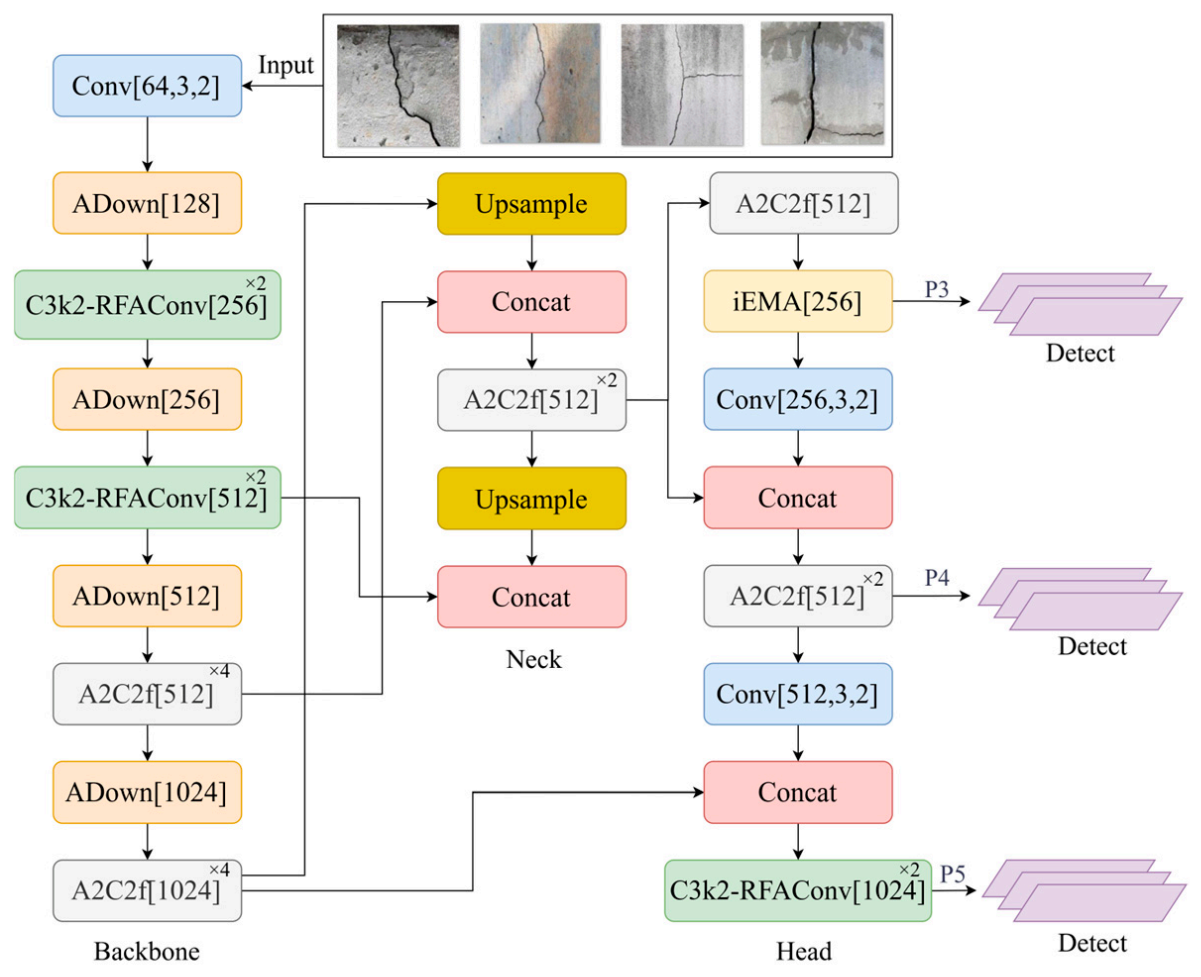
Overall architecture of MCL-YOLO.

However, in complex engineering environments, the original YOLOv12 architecture struggles with micro-cracks, low-contrast surfaces, non-uniform illumination, and noise, frequently leading to missed detections, inaccurate bounding-box localization, and false positives. These limitations stem from the rapid attenuation of edge responses during conventional downsampling, the lack of adaptability of fixed receptive-field convolutions to irregular crack morphologies, and insufficient focus on weak-texture regions within existing feature aggregation methods. To address these challenges, we propose the MCL-YOLO model (see Figure 2), which integrates ADown, C3k2-RFAConv, and iEMA as an architectural combination targeting information retention during downsampling, receptive-field feature weighting, and multi-scale semantic enhancement. Specifically, the ADown module uses multi-branch feature fusion with the design objective of retaining informative local responses and reducing information loss during early downsampling. The C3k2-RFAConv composite module applies the receptive-field attention-weighting mechanism of RFAConv to adaptively reweight spatial contributions within each local receptive field, with the aim of improving feature representation for curved and branched crack patterns. Furthermore, the iEMA module applies multi-scale spatial interaction with the aim of strengthening informative responses in weak-texture regions. This synergistic design effectively resolves the trade-off between detail preservation and model lightweighting, providing a high-precision and computationally efficient solution in bridge crack detection.

#### 3.2.1. iEMA Dynamic Feature Enhancement Module

**Figure 3:**
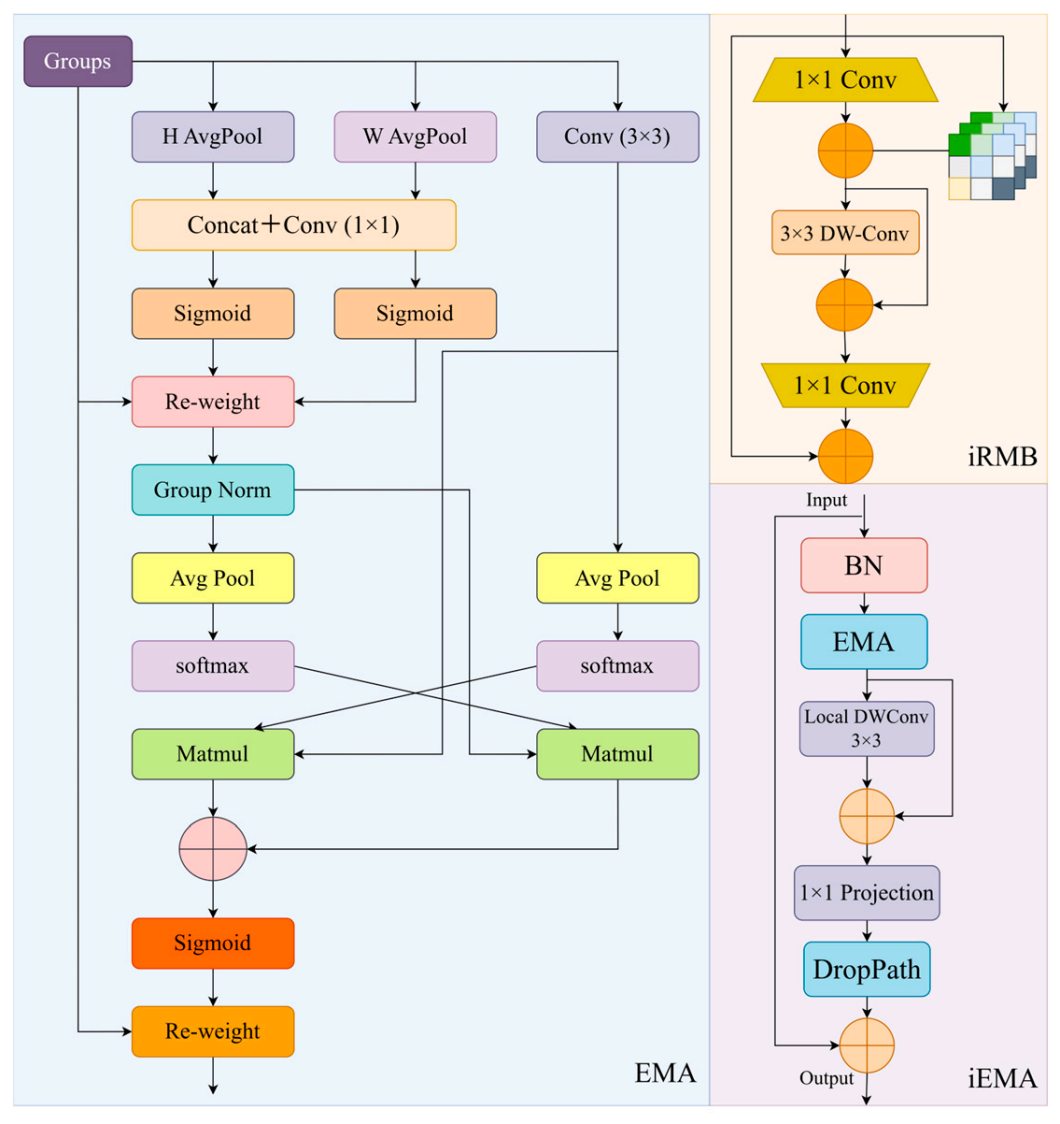
Architecture of the iEMA module.

To address the unstable crack feature response and the susceptibility of weak-texture information to background interference in complex scenarios, this study constructs an iEMA dynamic feature enhancement unit. The iEMA module combines the grouped cross-spatial weighting mechanism of EMA [14] with a lightweight local residual transformation inspired by iRMB [15]. It does not incorporate the complete iRMB block. Specifically, the window-based Q–K attention and value-projection branch of iRMB are omitted, while the 3 × 3 depthwise convolution, 1 × 1 output projection, and residual connections are retained for local feature refinement. Therefore, iEMA should be regarded as a lightweight hybrid module rather than a direct stacking of complete EMA and iRMB blocks. Through channel grouping, cross-spatial contextual modeling, and local residual compensation, it enables stable extraction and enhancement of fine-grained crack features. Figure 3 illustrates the overall architecture of the iEMA module. Given an input feature map *X*, the channels are first divided into *G* groups and rearranged into the batch dimension. This operation reduces the computational cost of feature interaction while preserving the semantic information within each channel group. The grouped features are subsequently normalized and processed by the EMA branch. By combining direction-aware spatial encoding with multi-scale feature interactions, the EMA branch generates input-adaptive attention weights that emphasize informative crack regions and suppress irrelevant background responses. This operation facilitates contextual modeling along elongated crack paths and improves the representation of weak boundaries and discontinuous crack segments. The attention-enhanced features are then passed through an iRMB-derived local residual transformation. In this collaborative architecture, the optimized EMA branch focuses on cross-spatial dependency modeling, while the iRMB residual path strengthens local structural details. Finally, the enhanced features are restored to the original channel dimension through a projection layer and connected with the input features through a residual connection. The computational process of iEMA is expressed as follows:

(1) $$X_{g}=R_{G}(X),X_{g}\in R^{\left(B\cdot G\right)\times (C/G)\times H\times W}$$

(2) $$X_{e}=\mathrm{EMA}\left(\mathrm{BN}\left(X_{g}\right)\right)⊙X_{g}$$

(3) $$Y=X+\mathrm{DropPath}\;\left(\mathrm{Proj}\left[R_{G}^{-1}\left(\varphi \left(X_{e}\right)\right)\right]\right)$$

where $X\in R^{B\times C\times H\times W}$ and $Y\in R^{B\times C\times H\times W}$ denote the input and output feature map, with *B*, *C*, *H* and *W* representing the batch size, number of channels, height, and width, respectively. *G* denotes the number of groups. $R_{G}\left(\cdot \right)$ and $R_{G}^{-1}\left(\cdot \right)$ denote the group-wise rearrangement and inverse rearrangement operations. $X_{g}$ and $X_{e}$ denote the rearranged grouped feature and the attention-enhanced grouped feature. $BN\left(\cdot \right)$ denotes batch normalization, and $EMA\left(\cdot \right)$ represents the cross-spatial multi-scale attention modeling process. ⊙ denotes element-wise multiplication, while $\varphi \left(\cdot \right)$ represents the iRMB-derived local residual feature transformation. $Proj\left(\cdot \right)$ denotes the channel projection operation, and $DropPath\left(\cdot \right)$ is used to enhance regularization during training.

#### 3.2.2. ADown Lightweight Downsampling Module

**Figure 4:**
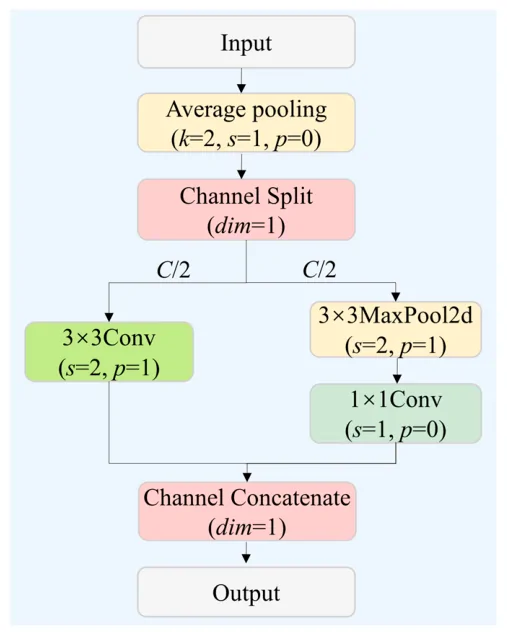
ADown lightweight downsampling module.

ADown is an existing lightweight composite downsampling unit originally introduced in YOLOv9 [12]. In this study, it is incorporated into the YOLOv12 architecture as a lightweight downsampling operator to reduce the computational cost associated with strided convolution and alleviate the loss of local details caused by single-path downsampling. Instead of directly performing strided convolution over the entire input feature map, ADown employs a channel-splitting strategy with two heterogeneous parallel branches. Convolution-based and pooling-based downsampling operations are applied to different feature subspaces, thereby producing complementary downsampled features while maintaining low computational complexity. As shown in Figure 4, the input feature map is first processed by average pooling with a kernel size of 2 × 2 and a stride of 1. This operation primarily smooths local responses rather than directly halving the spatial resolution. The resulting feature map is then evenly divided into two sub-features along the channel dimension. In the first branch, a 3 × 3 convolution with a stride of 2 reduces the spatial resolution while extracting discriminative local structural features. In the second branch, 3 × 3 max pooling with a stride of 2 is first applied to preserve locally salient responses, followed by a 1 × 1 convolution for channel projection. Finally, the outputs of the two branches are concatenated along the channel dimension to produce the downsampled feature map. Compared with directly applying a 3 × 3 strided convolution to all input channels, the ADown structure reduces the convolutional computation and enhances the diversity of the downsampled features through the complementary processing of the convolution and pooling branches. The computational process of ADown can be expressed as shown in Equations (4)–(6).

(4) $$X_{s}={AvgPool}_{k=2,s=1,p=0}(X)$$

(5) $$\left(X_{1},{\;X}_{2})={Split}_{C}(X_{s}\right)$$

(6) $$Y=Concat\left({Conv}_{3\times 3}(X_{1}),{Conv}_{1\times 1}\left({MaxPool}_{3\times 3}(X_{2})\right)\right)$$

where *X* denotes the input feature map, and $X_{s}$ represents the intermediate feature map obtained after average-pooling preprocessing. Split$\left(\cdot \right)$ denotes an equal partition along the channel dimension, while $X_{1}$ and $X_{2}$ represent the resulting channel-wise sub-features. Conv$\left(\cdot \right)$ denotes a convolutional unit consisting of convolution, batch normalization, and an activation function. Concat$\left(\cdot \right)$ denotes feature concatenation along the channel dimension, and *Y* represents the output feature map of the ADown module.

#### 3.2.3. C3k2-RFAConv Composite Feature Extraction Module

**Figure 5:**
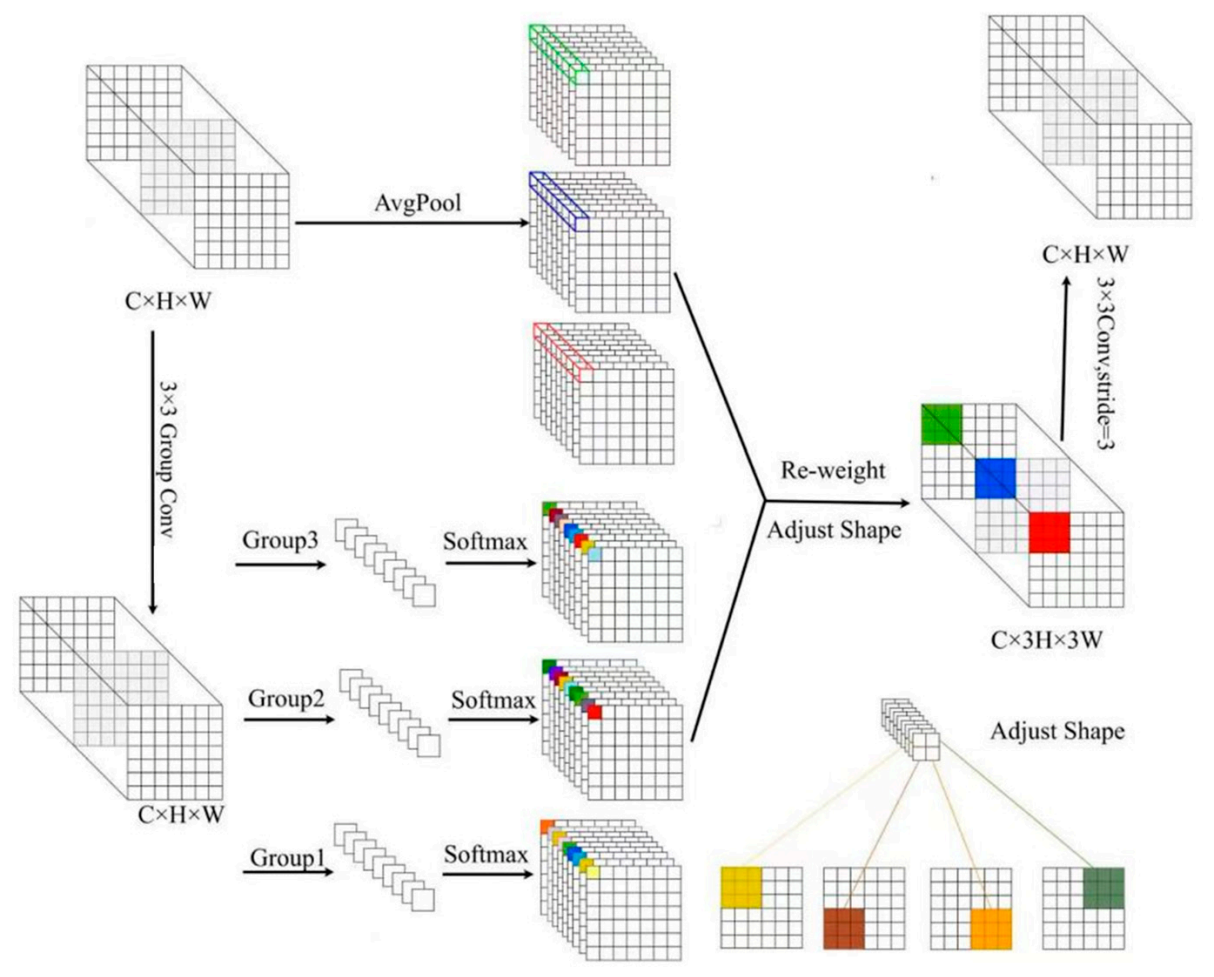
RFAConv module.

**Figure 6:**
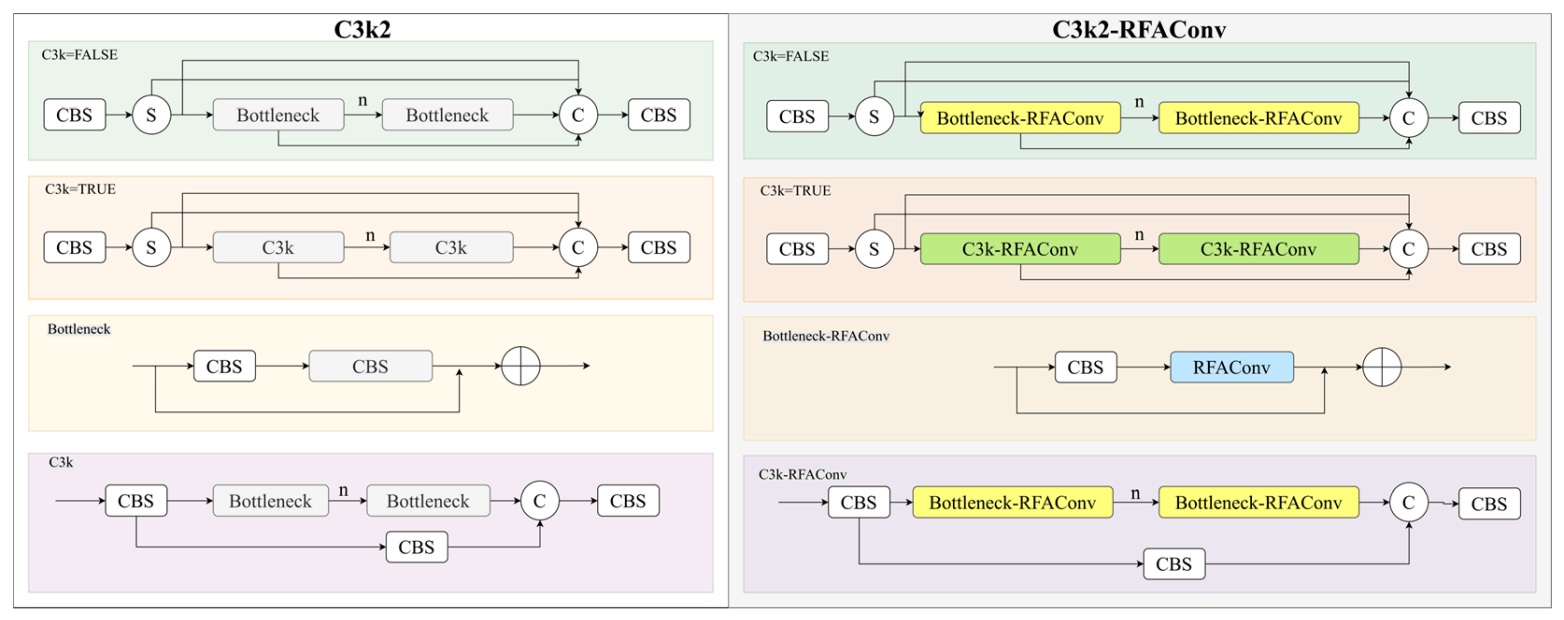
Structural comparison between the C3k2 and C3k2-RFAConv modules.

Bridge cracks generally exhibit complex geometric characteristics, including slenderness, curvature, branching, local widening, and texture discontinuity. The original C3k2 module achieves efficient parameter utilization and feature reuse through channel splitting, bottleneck stacking, cross-path connections, and feature concatenation. However, the internal Bottleneck still relies primarily on standard convolution for local feature extraction. Therefore, it is difficult to adaptively adjust receptive-field contributions according to crack trajectories, width variations, and differences in background textures. Receptive-Field Attention Convolution (RFAConv), as shown in Figure 5, operates on receptive-field spatial features and generates adaptive attention weights for individual feature elements within each local receptive field, thereby improving the sensitivity of convolution to spatial variations [16]. Based on these characteristics, RFAConv is embedded into the internal Bottleneck of C3k2 to construct the proposed C3k2-RFAConv module, as shown in Figure 6. Specifically, the original channel-splitting, cross-path connection, repeated-stacking, and feature-concatenation structures of C3k2 are retained, while the second standard convolution in each Bottleneck is replaced with RFAConv to form a Bottleneck-RFAConv unit. When C3k = True, the corresponding C3k-RFAConv unit is constructed using the modified Bottleneck-RFAConv units. After repeated stacking, the enhanced features are concatenated with the bypass features along the channel dimension and subsequently fused through the final convolutional layer. Unlike simply enlarging convolution kernels or stacking standard convolutions, this module emphasizes the adaptive selection of positional contributions within the receptive field. Specifically, RFAConv first constructs receptive-field units within local windows and uses attention weights to characterize the importance of different sampling positions. The weighted local features are then spatially rearranged and mapped through convolution, enabling the convolutional response to dynamically adjust according to crack morphology and background texture variations. After being embedded into C3k2, the proposed module retains the lightweight path reuse advantage of C3k2 while improving the model’s adaptability to complex crack morphologies, such as curved cracks, branched cracks, and cracks with varying widths.

## 4. Experimental Design

### 4.1. Experimental Setup

**Figure 7:**
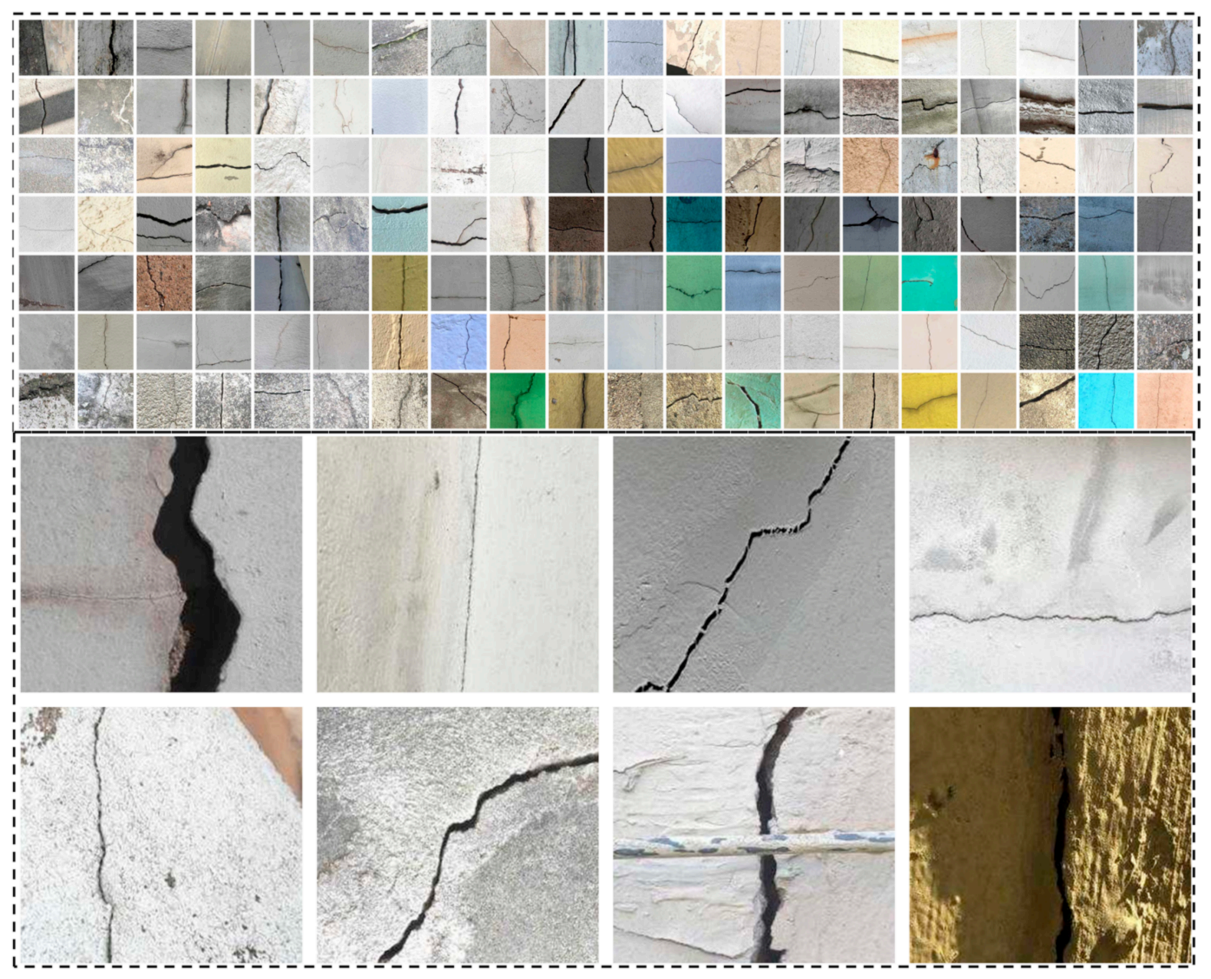
Examples of crack images in the dataset.

To improve experimental reproducibility and include diverse crack morphologies and background conditions in the evaluation, a bridge crack dataset comprising 4029 images and containing a single crack class was constructed. The dataset was commercially acquired from a third-party data provider. According to the acquisition information supplied by the provider, the images were independently captured from different positions and viewpoints during an on-site inspection of a bridge in Hong Kong, rather than being continuously extracted from video sequences or generated through densely overlapping image cropping. The collected images cover several bridge components, including bridge decks, piers, and abutments, and contain multiple crack morphologies and environmental interference factors [43]. These samples cover complex backgrounds, diverse crack morphologies, and ambiguous boundary characteristics observed in bridge images, including thin, wide, discontinuous, and multiple coexisting cracks, as well as common environmental interference such as strong or weak illumination, water stains, surface contamination, and shadows. Compared with datasets collected under relatively simple backgrounds and ideal imaging conditions, the dataset constructed in this study pays more attention to scale variations, morphological randomness, and boundary uncertainty of crack targets in complex backgrounds. Typical crack samples in the dataset are shown in Figure 7. All images were annotated with bounding boxes using LabelImg v1.8.6 and resized to 640 × 640 pixels during preprocessing. During annotation, spatially adjacent but visually separable independent cracks were assigned separate bounding boxes, whereas connected branches belonging to the same continuous crack were enclosed within a single bounding box. A long continuous crack was treated as one instance unless a clear physical discontinuity separated it from another crack. The annotations consist of rectangular bounding boxes rather than pixel-level crack masks; therefore, the task evaluates crack-instance detection and bounding-box localization rather than pixel-level crack-boundary delineation. The corresponding bounding-box coordinates were transformed accordingly to ensure consistency between the input dimensions and annotation information. For model training, parameter selection, and held-out performance evaluation within the constructed dataset, the images were randomly divided into training, validation, and test sets at a ratio of approximately 7:2:1, containing 2820, 805, and 404 images, respectively.

**Figure 8:**
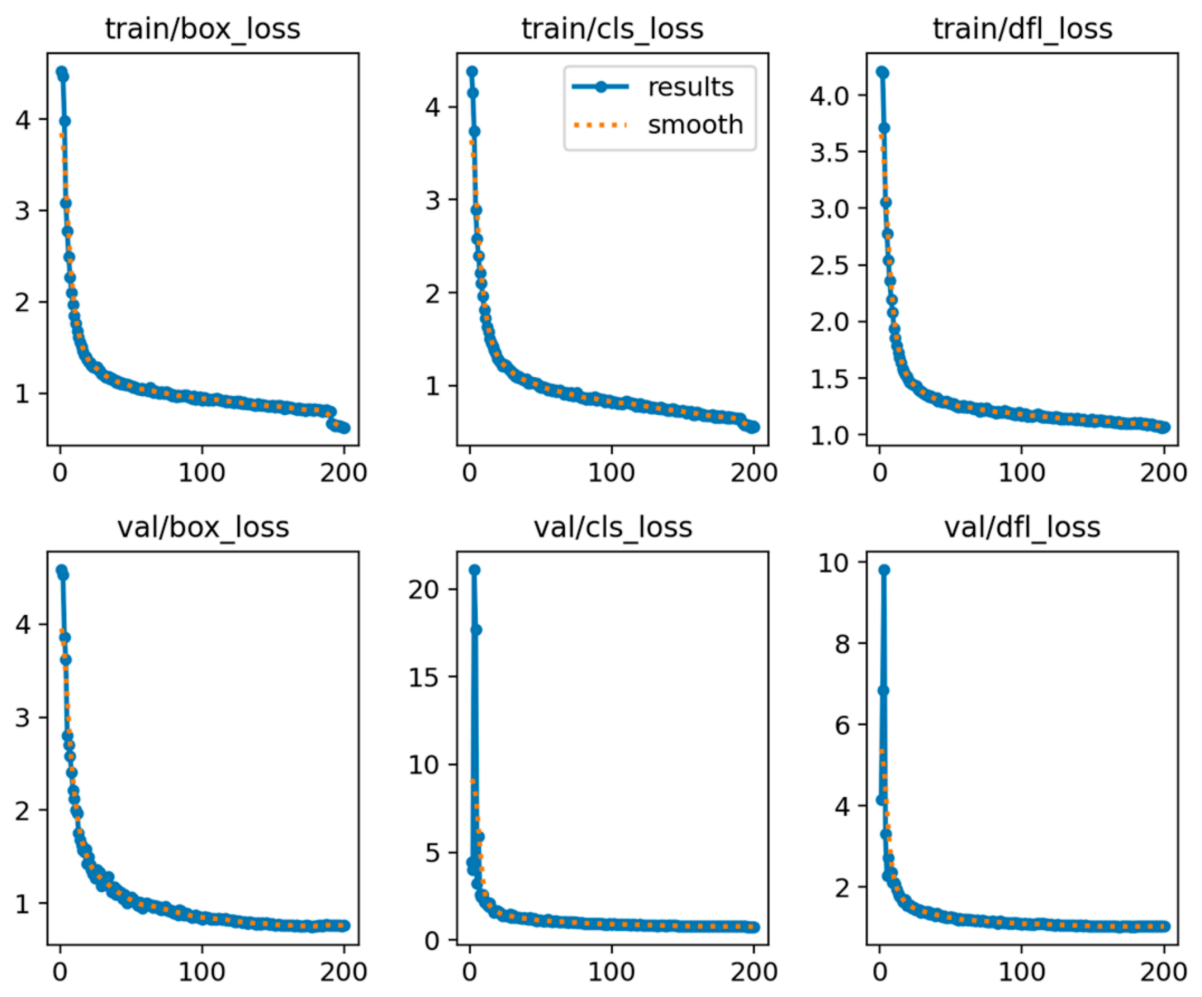
Training and validation loss curves.

As shown in Table 2, all models were trained and tested under the same hardware and software environments to ensure the comparability of the experimental results. The experimental platform was equipped with an NVIDIA GeForce RTX 4090 GPU with 24 GB of memory and an AMD EPYC 7513 32-Core Processor. The operating system was Ubuntu 22.04, and the software environment included Python 3.10.16, CUDA 12.4, PyTorch 2.5.1, and TorchVision 0.19.0. During model training, the stochastic gradient descent (SGD) optimizer was adopted. The number of training epochs was set to 200, and the batch size was set to 32. Mosaic data augmentation was introduced to improve the adaptability of the model to scale variations, target morphology differences, and complex background interference. To avoid ineffective iterations and reduce the risk of overfitting, an early-stopping patience value of 100 was used during training. As shown in Figure 8, the training and validation box_loss, cls_loss, and dfl_loss curves exhibited consistent downward trends and gradually converged as the number of training epochs increased, without evident late-stage divergence. These results indicate that the optimization process remained stable throughout training.

### 4.2. Evaluation Metrics

In this study, model performance was comprehensively evaluated in terms of detection accuracy, missed-detection control, bounding-box localization, and computational complexity. Precision (*P*), Recall (*R*), *mAP*@50, *mAP*@50:95, GFLOPs, and parameter count were selected as evaluation metrics. Precision represents the proportion of true crack instances among all instances predicted as cracks and primarily reflects the ability of the model to suppress false detections caused by background noise and crack-like interference. Recall represents the proportion of ground-truth crack instances correctly detected by the model, with a higher Recall value indicating a lower missed-detection rate. *mAP*@50 denotes the mean average precision across all classes at an Intersection over Union (IoU) threshold of 50% and is used to evaluate the overall detection performance. *mAP*@50:95 represents the mean average precision averaged over IoU thresholds ranging from 50% to 95%. Because IoU is calculated between the predicted and ground-truth bounding boxes, *mAP*@50:95 reflects detection performance across multiple bounding-box overlap thresholds and does not evaluate pixel-level crack-boundary delineation. GFLOPs is used to measure the computational complexity; a higher GFLOPs value indicates greater computational resource requirements. To complement GFLOPs, parameter count (Params) was additionally reported as an indicator of model complexity. The calculation formulas for evaluation metrics are as follows.

(7) $$P=\frac{TP}{TP+FP}$$

(8) $$R=\frac{TP}{TP+FN}$$

(9) $$AP=\int_{0}^{1}P(R)\;dR$$

(10) $$mAP=\frac{1}{N}\sum_{i=1}^{N}AP_{i}$$

where *TP* denotes the number of correctly detected crack targets, *FP* denotes the number of background or non-crack targets incorrectly detected as cracks, and *FN* denotes the number of crack targets missed by the model.

## 5. Discussion

### 5.1. Ablation Experiments

**Figure 9:**
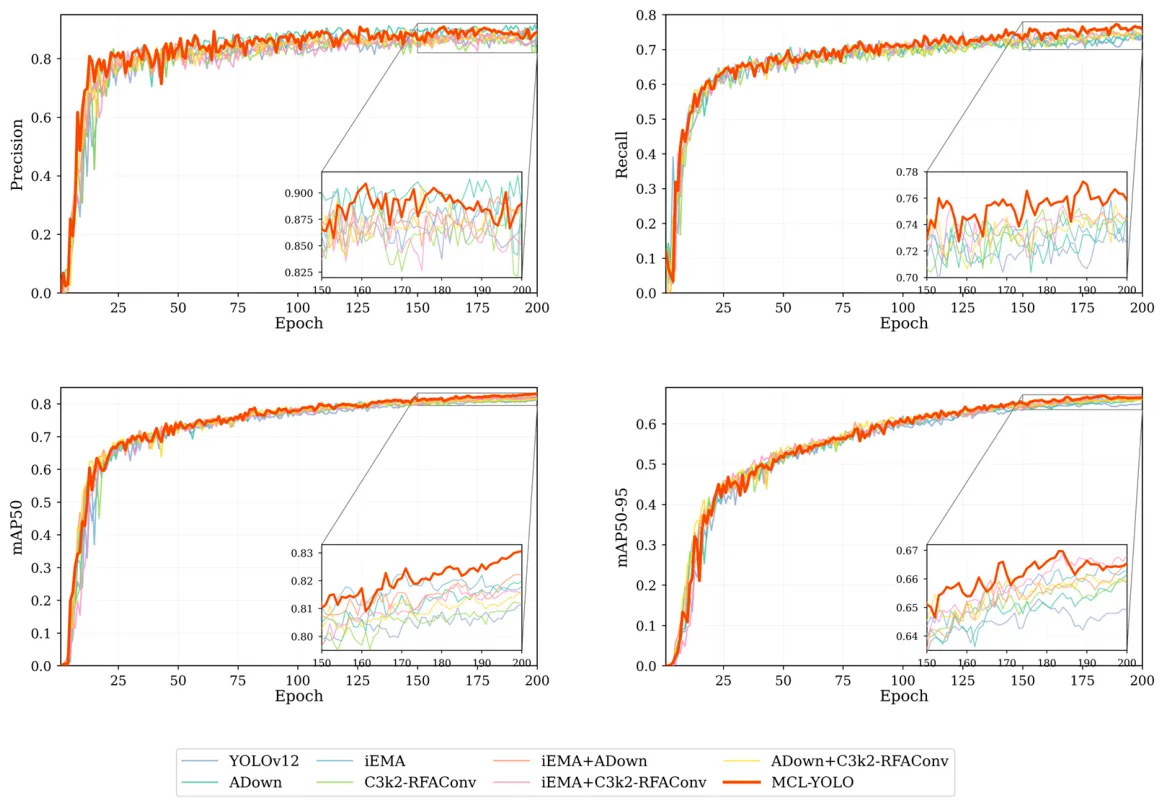
Changes in evaluation metrics of different ablation models during training.

**Figure 10:**
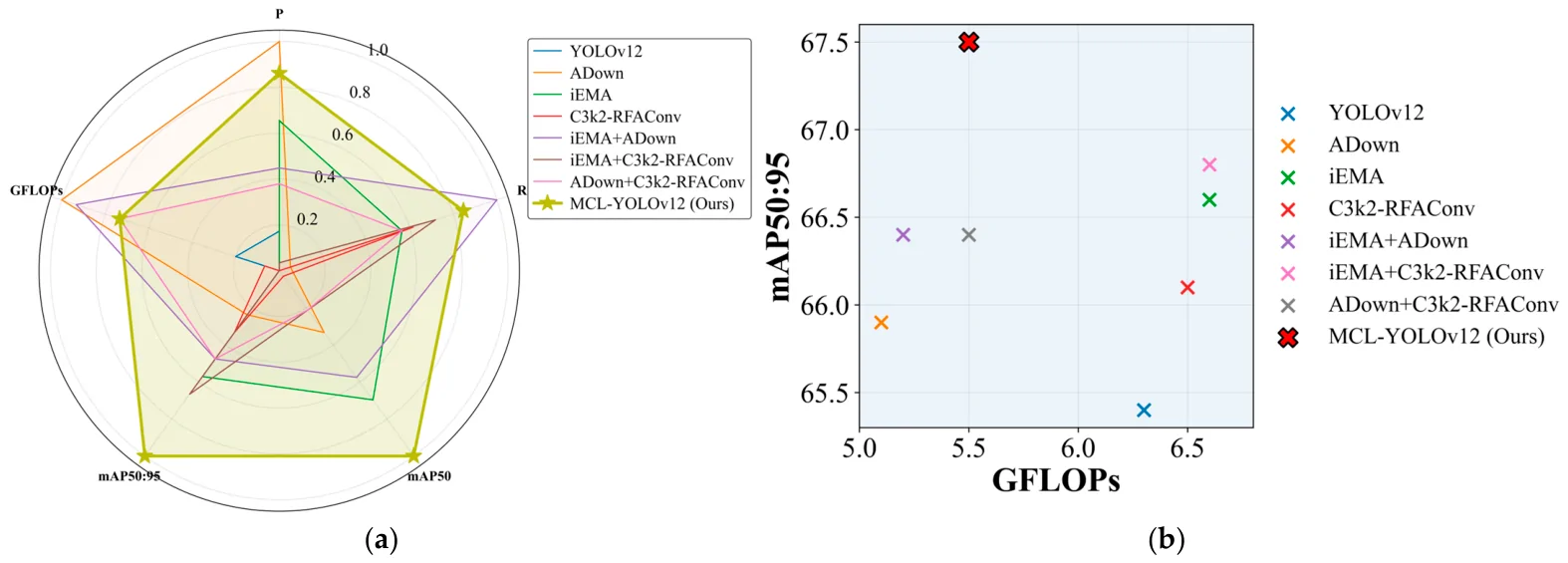
Comprehensive performance comparison of ablation models: (**a**) radar chart; (**b**) *mAP*@50:95-GFLOPs scatter plot.

To verify the effectiveness of ADown, iEMA, and C3k2-RFAConv modules for bridge crack detection, ablation experiments were conducted using the original YOLOv12 as the baseline under identical experimental settings. A progressive comparison scheme was established by introducing individual modules, pairwise combinations, and all three modules. The detailed results are presented in Table 3. Figure 9 illustrates the variation trends of Precision, Recall, *mAP*@50, and *mAP*@50:95 for different ablation models during training. The late-stage convergence interval is locally enlarged in the lower-right corner. It can be observed that all models show rapid improvement in the early training stage and then gradually enter a stable convergence stage. MCL-YOLO achieves a higher late-stage convergence level in both the *mAP*@50 and *mAP*@50:95 curves. Figure 10 further compares the overall performance of the ablation models through joint visualization of multiple metrics. In Figure 10a, a radar chart presents Precision, Recall, *mAP*@50, *mAP*@50:95, Params, and GFLOPs. The four accuracy-related metrics are positively normalized, whereas GFLOPs is inversely normalized. Therefore, a higher value in the radar chart indicates better predictive performance or lower computational cost. In Figure 10b, GFLOPs and *mAP*@50:95 are used as the horizontal and vertical axes, respectively, to construct a complexity–accuracy trade-off space for evaluating the balance between detection accuracy and computational cost among the different models.

In the single-module ablation analysis, after ADown was independently introduced into the baseline model, Params and GFLOPs decreased from 2.568 M and 6.3 to 2.086 M and 5.1, while Precision, *mAP*@50, and *mAP*@50:95 increased from 0.871, 0.811, and 0.654 to 0.895, 0.822, and 0.659, respectively. This indicates that ADown can not only reduce computational complexity but also improve the retention of key features during downsampling. It should be noted that although adown achieved the highest Precision, its Recall was only 0.726, suggesting that this module mainly contributes to reducing redundant computation and suppressing false detections, while its improvement in the recall of tiny, weak-texture, and discontinuous cracks is limited. When iEMA was introduced independently, all four detection metrics outperformed those of the baseline model. In particular, *mAP*@50:95 increased from 0.654 to 0.666, while the Params and GFLOPs increased moderately from 2.568 M and 6.3 to 2.661 M and 6.6. This result indicates that iEMA improves overall crack-feature representation on the current test set, thereby improving bounding-box localization performance across different IoU thresholds, at the cost of a modest increase in model complexity [40,41]. The C3k2-RFAConv module achieved a relatively high Recall of 0.748 among the single-module schemes, indicating that receptive-field attention weighting helps enhance feature representation within local receptive fields [16]. However, its Precision decreased to 0.866, suggesting that relying solely on receptive-field attention weighting is still insufficient for suppressing complex background interference.

The dual-module experiments demonstrate that the modules are complementary across different evaluation metrics. The iEMA+ADown configuration achieves the highest Recall of 0.763 among all tested configurations, while requiring only 5.2 GFLOPs. Compared with iEMA alone, the addition of ADown reduces GFLOPs from 6.6 to 5.2 and increases Recall from 0.746 to 0.763, although Precision, *mAP*@50, and *mAP*@50:95 decrease slightly from 0.885, 0.834, and 0.666 to 0.879, 0.830, and 0.664. This configuration therefore favors detection completeness and computational efficiency rather than maximizing overall detection accuracy. For the combinations involving C3k2-RFAConv, two distinct patterns can be observed. When combined with iEMA, all four detection metrics improve compared with C3k2-RFAConv alone, with Precision increasing from 0.866 to 0.867, Recall from 0.748 to 0.752, *mAP*@50 from 0.812 to 0.818, and *mAP*@50:95 from 0.661 to 0.668. The resulting *mAP*@50:95 of 0.668 is the highest among the dual-module configurations, while the computational cost increases only slightly from 6.5 to 6.6 GFLOPs. This result suggests that the combination of iEMA and C3k2-RFAConv is more favorable for improving detection performance under stricter bounding-box overlap criteria. In contrast, combining C3k2-RFAConv with ADown increases Precision from 0.866 to 0.877, *mAP*@50 from 0.812 to 0.818, and *mAP*@50:95 from 0.661 to 0.664, while Recall decreases only marginally from 0.748 to 0.746. Meanwhile, the parameter count decreases from 2.585 M to 2.235 M and GFLOPs from 6.5 to 5.5. Therefore, the ADown+C3k2-RFAConv configuration provides a more favorable balance between detection performance and computational complexity. The dual-module results indicate that the iEMA+ADown combination primarily improves Recall and computational efficiency, the iEMA+C3k2-RFAConv combination favors *mAP*@50:95, and the ADown+C3k2-RFAConv combination achieves a balanced improvement in detection performance while reducing model complexity. These results further support the complementary roles of the three modules and provide the basis for their joint integration in MCL-YOLO.

**Figure 11:**
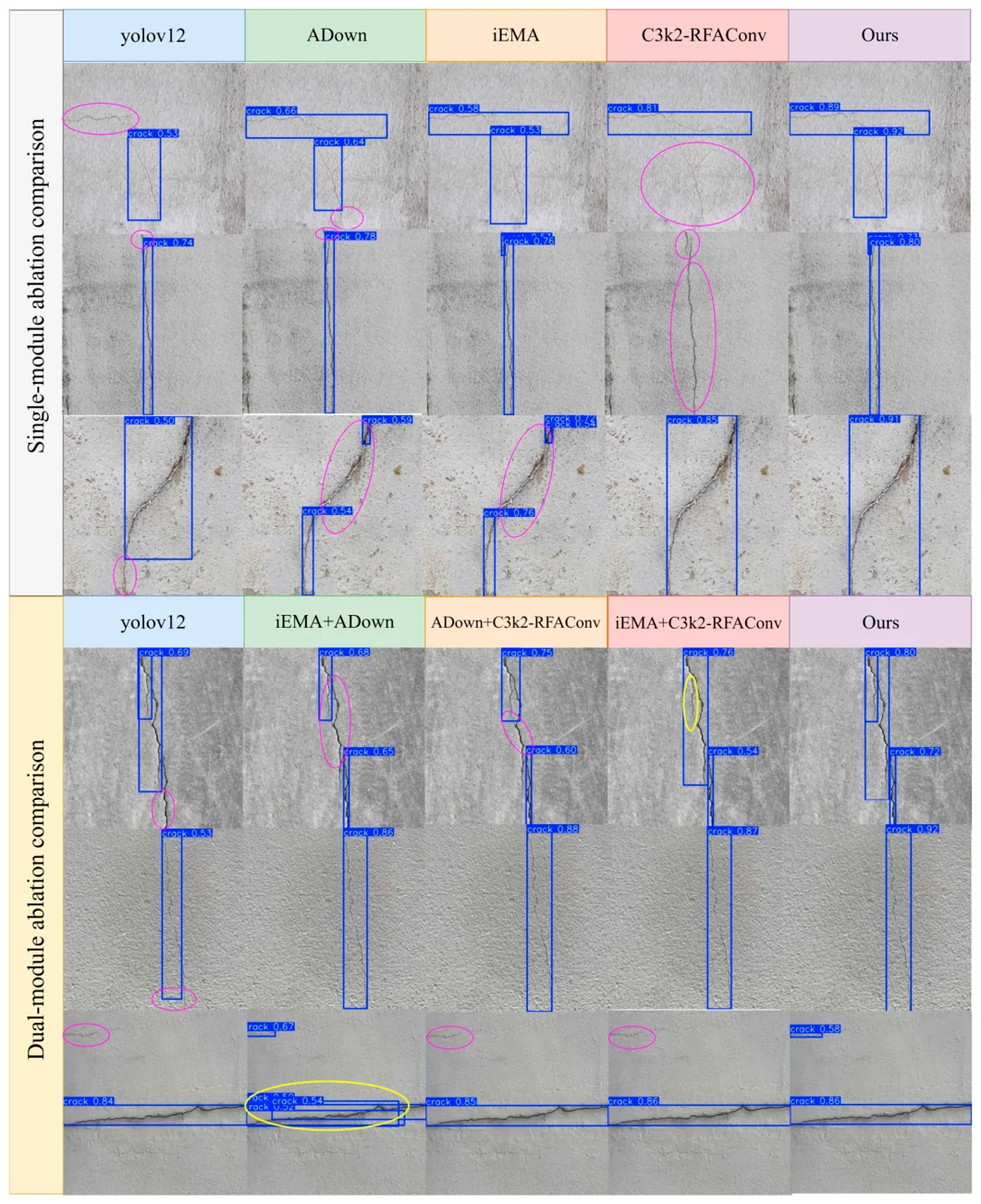
Visual comparison of crack detection results for different ablation models (magenta circles indicate missed-detection regions, and yellow circles indicate regions of target merging or split detection).

Although the Precision of the MCL-YOLO model (0.891) was slightly lower than that obtained using ADown alone (0.895), and its Recall (0.757) was slightly lower than that of the iEMA+ADown combination (0.763), it achieved the highest *mAP*@50 and *mAP*@50:95 values among all ablation variants. For bridge crack detection, the improvement in *mAP*@50:95 is particularly important because *mAP*@50:95 integrates the evaluation results over multiple IoU thresholds and reflects both detection reliability and bounding-box localization quality under stricter overlap criteria. As shown in Figure 10b, MCL-YOLO is located in the upper-left region of the *mAP*@50:95-GFLOPs space, achieving the highest *mAP*@50:95 at a relatively low theoretical arithmetic complexity. In addition, the complete model contains only 2.240 M parameters, which is lower than the 2.568 M parameters of the YOLOv12 baseline. This indicates that the performance improvement of the complete model is not achieved by simply increasing computational complexity. Instead, the three modules provide metric-dependent complementary effects, and their complete combination achieves the highest *mAP*@50 and *mAP*@50:95 on the current dataset, although it does not achieve the best result for every individual metric. To further assess the repeatability of the complete model, MCL-YOLO was independently trained two additional times under the same experimental configuration. The standard deviations of Precision, Recall, *mAP*@50, and *mAP*@50:95 obtained from the repeated experiments were 0.006, 0.007, 0.008, and 0.008, respectively. All four standard deviations remained below 0.01, providing additional evidence that the overall detection performance of the complete model is repeatable under the evaluated settings. As a result, the model achieves a better balance among performance under strict IoU criteria and computational complexity. Figure 11 presents the qualitative detection results of different ablation schemes on representative crack samples. In the figure, magenta circles mark missed-detection regions, including weak-texture, low-contrast, locally discontinuous, or fine cracks at crack ends. Yellow circles indicate target separation errors, including cases where adjacent independent cracks are incorrectly merged into a single detection box, or where a single continuous crack is redundantly split into multiple detection boxes. The comparison shows that the complete MCL-YOLO model can cover crack targets more completely when facing various challenging samples, while reducing missed detections, duplicate detections, and bounding-box fragmentation. These results further verify the effectiveness of the proposed module combination in complex bridge crack detection scenarios.

### 5.2. Comparative Experiments

To further evaluate the comprehensive performance of MCL-YOLO, this study selected the lightweight configurations YOLOv8n [44], YOLOv9t [12], YOLOv10n [45], YOLO11n [46], and YOLOv12 [13], together with ASF-YOLO [47], Hyper-YOLO [48], and GOLD-YOLO [49], as comparison models. YOLOv8, YOLO11 were implemented using their default n-scale configurations, while YOLOv9t represents the corresponding lightweight configuration of the YOLOv9 family. The ASF-YOLO baseline was implemented as a detection-only adaptation based on YOLO11n, in which two Zoom_cat modules and one ScalSeq module were incorporated into the neck for multi-scale feature fusion, while the original segmentation-specific branches were removed and the standard three-scale Detect head was retained for bounding-box prediction. All models were trained and evaluated using the same dataset split, with an input resolution of 640 × 640, 200 training epochs, a batch size of 32, the SGD optimizer, an initial learning rate of 0.01, and a weight decay of 0.0005. For each model, three independent training runs were conducted using three predefined random seeds, and the accuracy results reported in Table 3, Table 4 and Table 5 represent the averages of the three runs. The same pretrained initialization policy and data-augmentation settings were used, and no model-specific hyperparameter tuning was performed except for the architectural configurations required by the respective models. The comparison results are presented in Table 4. As shown in Table 4, compared with the YOLOv12n baseline, the Precision, Recall, *mAP*@50, and *mAP*@50:95 of MCL-YOLO increased from 0.871, 0.724, 0.811, and 0.654 to 0.891, 0.757, 0.844, and 0.675, respectively, corresponding to improvements of approximately 2.30%, 4.56%, 4.07%, and 3.21%. Meanwhile, the number of parameters and GFLOPs decreased from 2.568 M and 6.3 to 2.240 M and 5.5, corresponding to reductions of 12.77% and 12.70%. These results indicate that the performance improvement of MCL-YOLO does not rely on increased computational cost. Instead, the proposed model simultaneously improves detection accuracy and bounding-box localization quality while reducing computational overhead.

**Figure 12:**
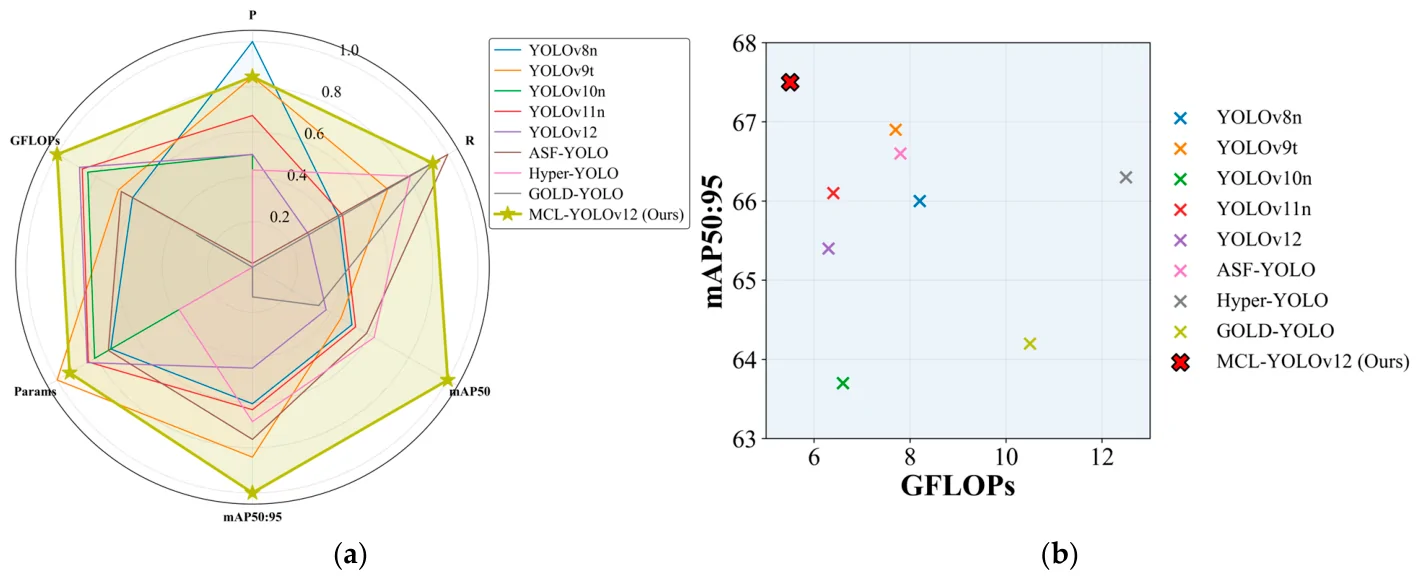
Comprehensive performance comparison of different models: (**a**) radar chart; (**b**) *mAP*@50:95-GFLOPs scatter plot.

It should be noted that some comparison models show certain advantages in individual metrics. YOLOv8n achieves the highest Precision of 0.900, indicating its strong ability to suppress false detections. ASFYOLO obtains the highest Recall of 0.761, suggesting stronger crack-recall capability. YOLOv9t has the smallest parameter count of 2.006 M while achieving an *mAP*@50:95 of 0.669, demonstrating a favorable balance between model compactness and high-threshold localization performance. However, these models still have limitations in terms of computational complexity or overall detection accuracy. In contrast, although MCL-YOLO does not achieve the highest values for Precision, Recall and Params as individual metrics, it simultaneously obtains the highest *mAP*@50, the highest *mAP*@50:95, and the lowest GFLOPs. Figure 12a further compares the overall performance of different models in terms of Precision, Recall, *mAP*@50, *mAP*@50:95, Params, and GFLOPs. It can be observed that MCL-YOLO maintains a high level across multiple dimensions and covers a larger area in the radar chart, indicating more balanced overall performance. Figure 12b further illustrates the relationship between *mAP*@50:95 and GFLOPs. MCL-YOLO is located in the favorable upper-left region of the complexity–accuracy space, simultaneously achieving the highest *mAP*@50:95 and the lowest GFLOPs. This result demonstrates that MCL-YOLO improves detection performance under strict IoU criteria while reducing theoretical computational complexity, thereby achieving a superior accuracy–complexity balance compared with the other models.

**Figure 13:**
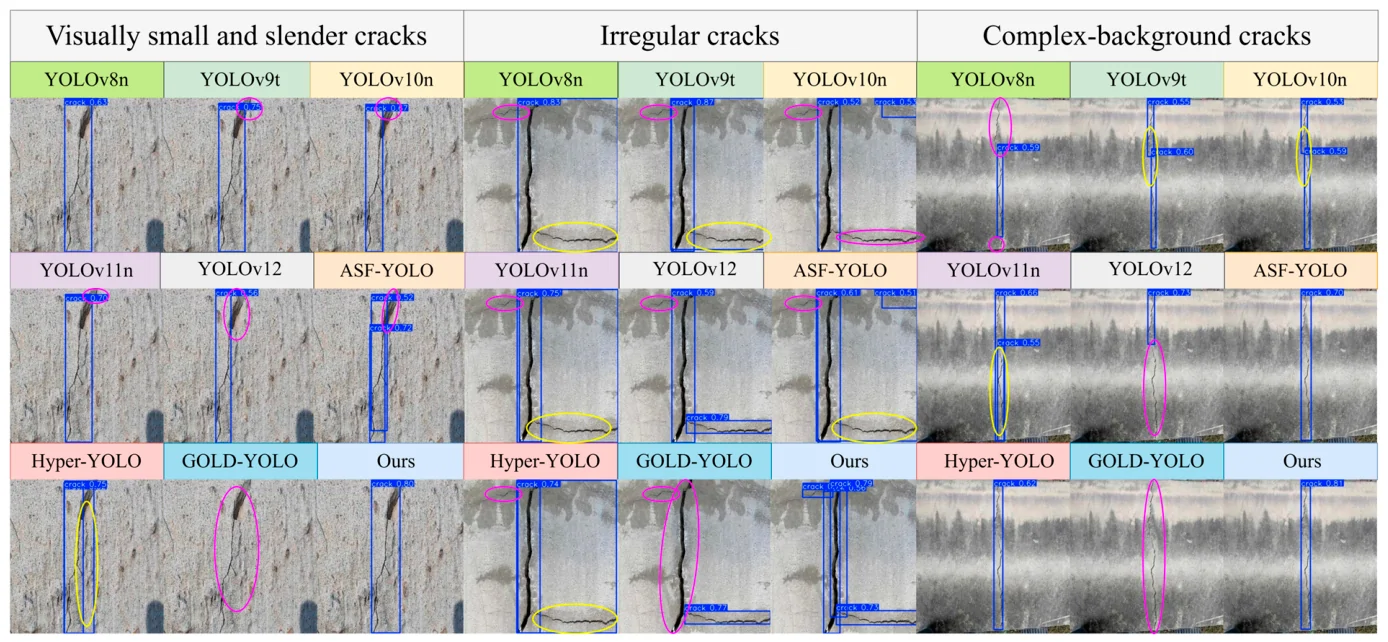
Visual comparison of crack detection results of different models (magenta circles indicate missed detections, and yellow circles indicate target merging or split-detection regions).

To complement the qualitative evaluation on the complete test set, the test images used for the scenario-based comparison were organized into three subgroups according to visually observable crack and background characteristics: visually small and slender crack, irregular cracks, and complex backgrounds cracks. Figure 13 further provides a qualitative comparison of the detection results of different models under three typical scenarios. All comparison models were evaluated using same images within each category. In the visually small and slender crack subgroup, some models can locate the main crack regions but may miss thin crack ends or low-contrast segments. In the irregular crack, crack curvature, branching, and adjacent cracks or similar textures may result in local missed detections or the incorrect merging of targets. In complex backgrounds, water stains, shadows, and rough concrete textures can cause false or missed detections. In contrast, MCL-YOLO generally shows more complete crack coverage, more stable detection confidence, and fewer target separation errors across the three types of scenarios. These visual results further complement and support the quantitative evaluation, providing additional evidence for the effectiveness of MCL-YOLO.

### 5.3. Comparative Experiments on Different Attention Mechanisms

To evaluate the implemented attention-enhanced configurations, comparative experiments were conducted using the same YOLOv12n baseline, dataset split, input resolution, and training settings. iEMA was compared with SimAM [50], EMA [14], iRMB [15], CoordAtt [51], MSDA [52], and SEAM [53]. The experimental results are shown in Table 5. As can be seen from Table 5, iEMA achieves the highest *mAP*@50 and *mAP*@50:95 values of 0.834 and 0.666, respectively, while maintaining a moderate model complexity of 2.661 M parameters and 6.6 GFLOPs. Although its parameter count is slightly higher than those of EMA, CoordAtt, and SimAM, it remains lower than those of iRMB, MSDA, and SEAM, indicating a favorable balance between detection performance and model complexity. In terms of Precision and Recall, iEMA achieves values of 0.885 and 0.746, respectively, both of which remain at relatively high levels. This suggests that the module achieves a favorable balance between false-detection suppression and missed-detection control. Although iRMB obtains the highest Precision of 0.894, its Recall is only 0.709, and its computational cost reaches 14.3 GFLOPs. This indicates that using iRMB alone tends to suppress false detections but weakens missed-detection control and the lightweight advantage of the model. EMA and CoordAtt show certain advantages in Recall; however, their *mAP*@50:95 values are 0.658 and 0.664, respectively, which are still lower than that of iEMA. This demonstrates that relying solely on channel or spatial positional attention is insufficient to fully improve bounding-box detection performance under stricter IoU thresholds in complex crack scenarios. MSDA and SEAM can enhance local feature responses or discriminative capability under occlusion scenarios, but their overall performance on the bridge crack dataset used in this study is inferior to that of iEMA. The iEMA module combines the cross-spatial feature-interaction capability of EMA with the local residual-modeling capability of iRMB. The observed improvements suggest that this design contributes to enhanced crack-feature representation and a favorable balance between detection accuracy and model complexity on the current bridge crack dataset.

## 6. Conclusions

Bridge cracks are important early indicators of structural service performance degradation. Timely and accurate identification of crack locations is of great significance for bridge health monitoring, damage early warning, and maintenance decision-making. To address the challenges of bridge crack targets in complex engineering environments, such as slender shapes, blurred boundaries, sparse textures, irregular morphologies, and strong background interference, this study proposes MCL-YOLO, a multi-module collaborative lightweight bridge crack detection model based on the YOLOv12 framework. The proposed model incorporates three functional modules, ADown, C3k2-RFAConv, and iEMA, to address the limitations of the original YOLOv12 in bridge crack detection by improving feature preservation during downsampling, adaptively reweighting spatial contributions within receptive fields, and enhancing discriminative semantic representations, respectively. The experimental results show that MCL-YOLO achieves Precision, Recall, *mAP*@50, and *mAP*@50:95 values of 0.891, 0.757, 0.844, and 0.675 on the test set, while maintaining a lightweight architecture with 5.5 GFLOPs and 2.240 M parameters. Compared with the original YOLOv12, MCL-YOLO achieves improved detection accuracy and localization performance with reduced computational complexity, demonstrating a favorable accuracy–efficiency trade-off. The model also shows promising potential for future deployment in resource-constrained inspection scenarios. Scenario-based qualitative comparisons further indicate that MCL-YOLO provides more complete detection responses under visually slender cracks, irregular crack patterns, and complex background conditions.

The ablation experiments further verified the effectiveness of each improved module. ADown reduced the computational complexity from 6.3 to 5.1 GFLOPs while modestly improving Precision, *mAP*@50, and *mAP*@50:95, demonstrating that its heterogeneous dual-branch downsampling design improves computational efficiency without degrading detection performance. iEMA achieved the highest *mAP*@50 and *mAP*@50:95 among the single-module variants, together with relatively high Precision and Recall, indicating a favorable balance between false-positive control and detection completeness. The C3k2-RFAConv module achieved relatively high Recall, indicating that receptive-field attention weighting contributes to enhanced feature representation and improved detection capability under the current experimental setting. The dual-module combination experiments show that ADown, iEMA, and C3k2-RFAConv exhibit complementary effects across different evaluation metrics, with iEMA+ADown favoring Recall and computational efficiency, iEMA+C3k2-RFAConv achieving the highest *mAP*@50:95 among the dual-module variants, and ADown+C3k2-RFAConv providing a favorable balance between detection performance and computational complexity. Although the complete model does not achieve the highest values in all individual metrics, it performs best in overall metrics such as *mAP*@50, *mAP*@50:95, and GFLOPs. This indicates that it achieves a favorable balance among detection accuracy, bounding-box localization quality, and computational efficiency. Comparative experiments with YOLOv8n, YOLOv9t, YOLOv10n, YOLO11n, YOLOv12n, ASF-YOLO, Hyper-YOLO, and GOLD-YOLO show that MCL-YOLO achieves the highest *mAP*@50 and *mAP*@50:95 while maintaining the lowest GFLOPs. This demonstrates that the proposed model not only improves crack detection accuracy under conventional IoU thresholds but also maintains strong bounding-box prediction capability under stricter localization evaluation conditions. The qualitative detection results further show that MCL-YOLO can reduce missed detections and target split-detection errors in scenarios involving low contrast, weak textures, local discontinuity, and complex background interference. MCL-YOLO also shows improved bounding-box localization of the visible crack instances. In summary, the proposed MCL-YOLO provides an improved solution for intelligent bridge crack detection in complex engineering environments, achieving a favorable balance among accuracy and efficiency.

Although the proposed MCL-YOLO achieves favorable experimental performance in bridge crack detection, several aspects still require further improvement. First, although the dataset used in this study covers various bridge components, crack morphologies, and environmental interference factors, the sample sources remain somewhat limited. Actual bridge inspection environments are more complex, and crack images may vary considerably across different regions, bridge types, material surfaces, imaging devices, and climatic conditions. Therefore, the generalization capability of the model under cross-region, cross-device, and cross-scenario conditions still needs further verification. Future work will expand the dataset by incorporating more real inspection images from different bridges, regions, and acquisition devices, and will evaluate model robustness through external datasets and controlled experiments involving illumination variation, noise, blur, and surface contamination. Furthermore, cross-domain transfer learning and self-supervised learning strategies will be explored to improve model adaptability under diverse inspection conditions. Second, this study focuses primarily on bounding-box-based crack detection, and quantitative assessment of crack geometry and damage severity, including crack width, length, orientation, and structural damage level, has not been further investigated. Although the qualitative results provide scenario-based evidence of improved detection behavior for visually small and slender cracks, the current dataset does not contain quantitative crack-size annotations or pixel-level masks. Therefore, size-specific detection performance and detailed crack characterization remain to be further studied. Future research will integrate pixel-level crack segmentation, width-based classification criteria, and three-dimensional reconstruction techniques to establish a complete framework from crack identification to quantitative damage assessment. Third, although the proposed modules improve overall detection performance, the current experiments mainly evaluate their effectiveness through overall detection metrics and qualitative comparisons. The specific contributions of each module to edge preservation, spatial feature refinement, and weak-texture representation have not been quantitatively isolated. Future work will introduce feature-response visualization, attention analysis, edge-preservation metrics, and morphology-specific subgroup evaluations to further investigate the underlying mechanisms of the proposed modules. In addition, although MCL-YOLO demonstrates reduced computational complexity and favorable inference efficiency on GPU hardware, direct deployment validation on representative edge platforms, such as embedded GPUs or mobile devices, remains necessary. Future studies will further evaluate end-to-end latency, energy consumption, and deployment stability under practical edge-computing environments.

## Figures and Tables

**Table 1:** Evolution of the YOLO series frameworks.

Release	Year	Primary Task	Contributions
YOLO	2015	Object Detection	Single-stage end-to-end detector
YOLOv2	2016	Object Detection	Batch normalization, dimension clustering, multi-scale training
YOLOv3	2018	Object Detection	Darknet-53 backbone, multi-scale prediction
YOLOv4	2020	Object Detection	CSPDarknet53 backbone, bag-of-freebies and bag-of-specials
YOLOv5	2020	Object Detection	Practical PyTorch implementation, CSP-PAN design
YOLOv6	2022	Object Detection	EfficientRep backbone, Rep-PAN, industrial deployment orientation
YOLOv7	2022	Object Detection	E-ELAN and trainable bag-of-freebies
YOLOv8	2023	Multi-task vision	Anchor-free head, decoupled design, unified multi-task framework
YOLOv9	2024	Object Detection	PGI and GELAN
YOLOv10	2024	Object Detection	Consistent dual assignments, NMS-free training
YOLOv11	2024	Multi-task vision	Improved efficiency and broader task support
YOLOv12	2025	Object Detection	Attention-centric design, R-ELAN and FlashAttention-based acceleration

**Table 2:** Training environment and parameter settings.

Parameter Name	Value	Parameter Name	Value
GPU	NVIDIA GeForce RTX 4090	Imgsz	640
GPU Memory	24 GB	Epochs	200
Operating System	Ubuntu 22.04	Batch Size	32
CPU	AMD EPYC 7513 32-Core Processor	Optimizer	SGD
Python Version	3.10.16	Workers	2
CUDA Version	12.4	Cache	FALSE
PyTorch Version	2.5.1	Patience	100
TorchVision	0.19.0	Data Augmentation	Mosaic

**Table 3:** Ablation experiment results.

Model	*P*	*R*	*mAP*50	*mAP*50:95	Params	GFLOPs
YOLOv12	0.871	0.724	0.811	0.654	2.568	6.3
ADown	**0.895**	0.726	0.822	0.659	**2.086**	**5.1**
iEMA	0.885	0.746	0.834	0.666	2.661	6.6
C3k2-RFAConv	0.866	0.748	0.812	0.661	2.585	6.5
iEMA + ADown	0.879	**0.763**	0.830	0.664	2.091	5.2
iEMA + C3k2-RFAConv	0.867	0.752	0.818	0.668	2.590	6.6
Adown + C3k2-RFAConv	0.877	0.746	0.818	0.664	2.235	5.5
MCL-YOLO	0.891	0.757	**0.844**	**0.675**	2.240	5.5

**Table 4:** Comparison results of different detection models.

Model	*P*	*R*	*mAP*50	*mAP*50:95	Params	GFLOPs
YOLOv8n	**0.900**	0.732	0.818	0.660	3.011	8.2
YOLOv9t	0.891	0.745	0.815	0.669	**2.006**	7.7
YOLOv10n	0.871	0.709	0.791	0.637	2.707	6.6
YOLO11n	0.881	0.733	0.819	0.661	2.590	6.4
YOLOv12	0.871	0.724	0.811	0.654	2.568	6.3
ASF-YOLO	0.843	**0.761**	0.822	0.666	2.965	7.8
Hyper-YOLO	0.867	0.751	0.824	0.663	4.290	12.5
GOLD-YOLO	0.842	0.757	0.809	0.642	5.662	10.5
MCL-YOLO (Ours)	0.891	0.757	**0.844**	**0.675**	2.240	**5.5**

**Table 5:** Comparison results of different attention mechanisms.

Model	*P*	*R*	*mAP*50	*mAP*50:95	Params	GFLOPs
SimAM	0.873	0.731	0.812	0.656	2.585	**6.5**
EMA	0.860	0.743	0.816	0.658	**2.568**	**6.5**
iRMB	**0.894**	0.709	0.811	0.653	2.850	14.3
CoordAtt	0.884	0.724	0.804	0.645	2.580	**6.5**
MSDA	0.871	0.729	0.804	0.655	2.914	7.1
SEAM	0.867	0.756	0.812	0.650	2.672	6.6
iEMA	0.885	**0.746**	**0.834**	**0.666**	2.661	6.6

## Data Availability

The dataset used in this study was commercially acquired from a third-party data provider and cannot be publicly redistributed due to licensing restrictions. Further information regarding the dataset may be obtained from the corresponding author upon reasonable request.
